# Metformin blocks progression of obesity-activated thyroid cancer in a mouse model

**DOI:** 10.18632/oncotarget.8989

**Published:** 2016-04-26

**Authors:** Jeongwon Park, Won Gu Kim, Li Zhao, Keisuke Enomoto, Mark Willingham, Sheue-Yann Cheng

**Affiliations:** ^1^ Laboratory of Molecular Biology, Center for Cancer Research, National Cancer Institute, National Institutes of Health, Bethesda, MD 20892, USA; ^2^ Current Address: Division of Endocrinology, Department of Internal Medicine Asan Medical Center, University of Ulsan College of Medicine, Songpa-gu, South Korea, Seoul 138-736

**Keywords:** thyroid carcinogenesis, obesity, metformin, mouse models

## Abstract

Compelling epidemiologic evidence indicates that obesity is associated with a high risk of human malignancies, including thyroid cancer. We previously demonstrated that a high fat diet (HFD) effectively induces the obese phenotype in a mouse model of aggressive follicular thyroid cancer (*Thrb^PV/PV^Pten*^+/−^mice). We showed that HFD promotes cancer progression through aberrant activation of the leptin-JAK2-STAT3 signaling pathway. HFD-promoted thyroid cancer progression allowed us to test other molecular targets for therapeutic opportunity for obesity-induced thyroid cancer. Metformin is a widely used drug to treat patients with type II diabetes. It has been shown to reduce incidences of neoplastic diseases and cancer mortality in type II diabetes patients. The present study aimed to test whether metformin could be a therapeutic for obesity-activated thyroid cancer. *Thrb^PV/PV^Pten*^+/−^mice were fed HFD together with metformin or vehicle-only, as controls, for 20 weeks. While HFD-*Thrb^PV/PV^Pten*^+/−^mice had shorter survival than LFD-treated mice, metformin had no effects on the survival of HFD-*Thrb^PV/PV^Pten*^+/−^mice. Remarkably, metformin markedly decreased occurrence of capsular invasion and completely blocked vascular invasion and anaplasia in HFD-*Thrb^PV/PV^Pten*^+/−^mice without affecting thyroid tumor growth. The impeded cancer progression was due to the inhibitory effect of metformin on STAT3-ERK-vimentin and fibronectin-integrin signaling to decrease tumor cell invasion and de-differentiation. The present studies provide additional molecular evidence to support the link between obesity and thyroid cancer risk. Importantly, our findings suggest that metformin could be used as an adjuvant in combination with antiproliferative modalities to improve the outcome of patients with obesity-activated thyroid cancer.

## INTRODUCTION

The incidence of thyroid cancer, the most common malignancy in endocrine organs, has been increasing rapidly in the past decades [1, 2]. At the same time, the rates of obesity and metabolic syndrome have also risen. Recent epidemiologic studies have shown a positive association of obesity with thyroid cancer incidence [3–6]. Many retrospective studies of patients with papillary thyroid cancer (PTC) show that a higher body mass index is correlated with a more aggressive PTC phenotype, such as increased tumor size, extrathyroidal invasion, and advanced tumor, node, metastasis (TNM) stage independent of age, sex, and other confounding factors [7]. These compelling epidemiologic data on the positive correlation of obesity with the risk of thyroid cancer prompted us to explore the molecular basis underpinning such a correlation.

We used a mouse model of follicular thyroid cancer (*Thrb^PV/PV^Pten*^+/−^mice) to elucidate the underlying mechanisms. *Thrb^PV/PV^Pten*^+/−^mice express a potent dominantly negative thyroid hormone receptor β (TRβPV) and haplo insufficiency in the *Pten* gene (phosphatase and tensin homologue deleted from chromosome 10) [8]. We fed *Thrb^PV/PV^Pten*^+/−^mice a high fat diet (HFD) to induce obesity marked by increased body weight, enlarged fat cells, and elevated serum leptin levels [9]. Biochemical and histopathologic analyses showed that the obese *Thrb^PV/PV^Pten*^+/−^mice exhibit more aggressive tumor progression with increased tumor cell proliferation, shortened survival, and frequent occurrence of anaplasia [9]. Moreover, we also identified leptin-JAK2-STAT3 signaling as one pathway that mediates the obesity-induced aggressive tumor progression. Thus, these findings not only provide direct molecular evidence to support the link between obesity and thyroid cancer risk, but also open the possibility of using *Thrb^PV/PV^Pten*^+/−^mice to test potential molecular targets for treatment of obesity-induced thyroid cancer.

More recently, we treated *Thrb^PV/PV^Pten*^+/−^mice with a STAT3-specific inhibitor, S3I-201, aiming to block the STAT3-downstream signaling to delay obesity-exacerbated thyroid cancer progression [10]. We found that S3I-201 effectively inhibits HFD-induced aberrant activation of STAT3 and its downstream targets to markedly inhibit thyroid tumor growth and prolong survival. S3I-201 also acts to decrease expression of the key regulators of the epithelial-mesenchymal-transition, i.e., vimentin and matrix metallo proteinases, to block anaplasia and lung metastasis [10]. Thus, using HFD-*Thrb^PV/PV^Pten*^+/−^mice, we have shown that inhibition of the STAT3 activity would be a novel treatment strategy for obesity-induced thyroid cancer.

With the availability of HFD-*Thrb^PV/PV^Pten*^+/−^mice as a preclinical mouse model, we expanded the search for other potential treatment modalities for obesity-induced thyroid cancer. We considered metformin (1,1-dimethylbiguanide hydrochloride), the most widely used antihyperglycemic drug for treatment of type II diabetes patient sowing to its effectiveness, safety profile, and affordability [11, 12]. In addition to its anti-diabetic effect, epidemiologic evidence suggests that metformin may lower cancer risk, increase healthy life span and improve outcomes among diabetic patients [13–19]. Numerous studies have shown that metformin could reduce the risk of developing solid tumors [20], such as colorectal, liver, pancreatic, stomach, breast, and thyroid cancer [20–24]. Recently, several studies have reported that metformin inhibits cell proliferation in thyroid cancer cells including medullary, anaplastic, and PTC cell lines [20, 25–27]. Still lacking, however, is direct molecular evidence to demonstrate the effectiveness of metformin in the treatment of obesity-induced thyroid cancer *in vivo.* In the present studies, we treated HFD-*Thrb^PV/PV^Pten*^+/−^mice with metformin and evaluated its effects on survival, tumor growth, tumor cell invasion, and occurrence of anaplasia. We found that metformin markedly decreased occurrence of capsular invasion and completely blocked vascular invasion and anaplasia in HFD-*Thrb^PV/PV^Pten*^+/−^mice. These results suggest that metformin could be beneficial for patients with obesity-activated thyroid cancer.

## RESULTS

### Metformin delays thyroid tumor progression in Thrb^PV/PV^Pten^+/−^mice with HFD-induced obesity

**Figure 1:**
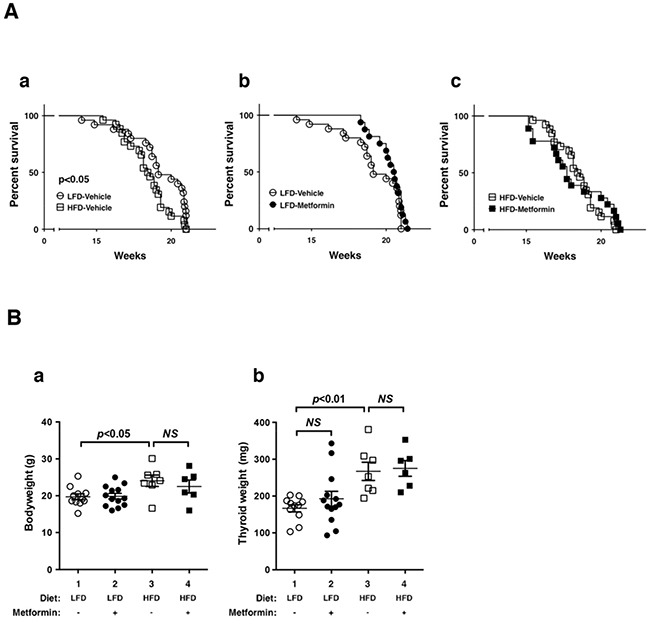
Effects of metformin on survival and thyroid tumor growth of LFD- or HFD- *Thrb^PV/PV^ Pten*^+/−^mice **A.** Survival curves for LFD versus HFD (n=25-26): (a) vehicle versus metformin (n=16-25) in LFD-treated mice and (b) vehicle versus metformin (n=18-26) in HFD-treated *Thrb^PV/PV^Pten*^+/−^mice (metformin, 100 mg/kg body weight). Data are presented by Kaplan-Meir methods and analyzed by log-rank test. **B.** Body weight (a) and thyroid weight (b) of vehicle-treated or metformin-treated LFD-*Thrb^PV/PV^Pten*^+/−^mice (n=11-13, age of 20-21 weeks) or HFD-*Thrb^PV/PV^Pten*^+/−^mice (n=6-7, age of 20-21 weeks). The p values are indicated.

Previously, we reported that the diet-induced obesity in *Thrb^PV/PV^Pten*^+/−^mice promoted thyroid carcinogenesis by reducing survival rate, increasing tumor growth, and advancing tumor stages [9]. To evaluate the effect of metformin on obesity-activated thyroid carcinogenesis, we analyzed survival, body weight, and thyroid weight in low fat diet (LFD)- or HFD-*Thrb^PV/PV^Pten*^+/−^mice. Figure 1A-a shows that the survival of HFD-*Thrb^PV/PV^Pten*^+/−^mice was significantly shorter than LFD-*Thrb^PV/PV^Pten*^+/−^mice. However, metformin had no apparent effect on the survival of LFD-*Thrb^PV/PV^Pten*^+/−^mice (Figure 1A-b) or HFD-*Thrb^PV/PV^Pten*^+/−^mice (Figure 1A-c). Consistent with our previous findings [9], the body weight of HFD-*Thrb^PV/PV^Pten*^+/−^mice was significantly higher than LFD-*Thrb^PV/PV^Pten*^+/−^mice (Figure 1B-a, data set 1 *vs* data set 3, n=7-11). However, metformin had no significant effect on the body weight of LFD- and HFD-*Thrb^PV/PV^Pten*^+/−^mice (Figure 1B-a). We next evaluated the effect of metformin on thyroid tumor growth. As reported previously [9], the thyroid weight of HFD-*Thrb^PV/PV^Pten*^+/−^mice was clearly higher than LFD-*Thrb^PV/PV^Pten*^+/−^mice (Figure 1B-b,data set 1 *vs* data set 3, n=7-11). However, metformin had no significant effect on the thyroid tumor growth of LFD-*Thrb^PV/PV^Pten*^+/−^mice (Figure 1B-b, data set 1 *vs* data set 2, n=11-13), and HFD-*Thrb^PV/PV^Pten*^+/−^mice (Figure 1B-b, data set 4 *vs* data set 3, n=6-7).

**Figure 2:**
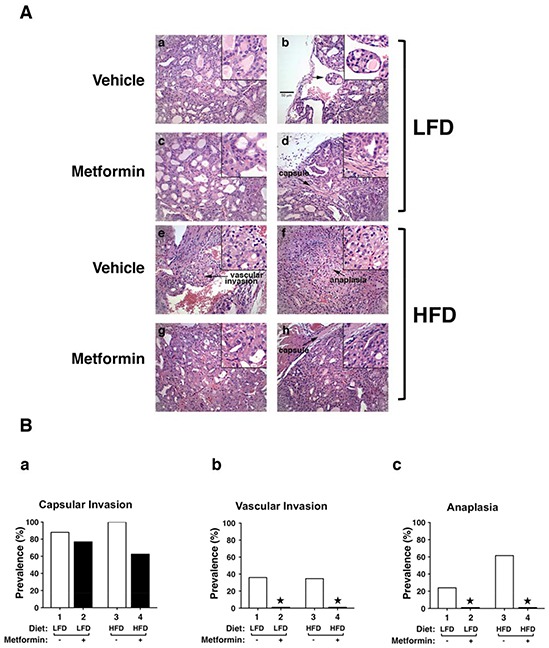
Effects of metformin on thyroid cancer progression of LFD- or HFD- *Thrb^PV/PV^ Pten*^+/−^mice **A.** Representative examples of hematoxylin and eosin (H&E)-stained thyroid sections from the vehicle-treated LFD (panels a and b), metformin-treated LFD (panel c and d), vehicle-treated HFD (panels e and f), metformin-treated HFD (panels g and h) groups of *Thrb^PV/PV^Pten*^+/−^mice. The magnification is X166. Arrows indicate pathological features of vascular invasion (panel e), capsular invasion (panels d and h) and anaplasia (panel f). The detailed pathological features are enlarged in a higher magnification of X332. **B.** Pathologic analysis of the vehicle-treated LFD (n=25), metformin-treated LFD (n=13), vehicle-treated HFD (n=26), and metformin-treated HFD (n=18) groups of *Thrb^PV/PV^ Pten*^+/−^mice. The prevalence of each pathologic feature in mice treated with vehicle or metformin is shown as percentage of occurrence for capsular invasion (panel a), vascular invasion (panel b) and anaplasia (panel c). *Represents no occurrence.

We further evaluated the effect of metformin on thyroid tumor progression by comparing histopathologic characteristics of *Thrb^PV/PV^Pten*^+/−^mice with different treatments at the same age. Consistent with our previous observations, HFD promoted tumor progression from extensive hyperplasia (Figure 2A, panel a) and early vascular invasion (panel b) in the thyroid of LFD*-Thrb^PV/PV^Pten*^+/−^mice to the more advanced stages of vascular invasion (panel e) and anaplasia (panel f). The frequency of occurrence of such pathologic changes promoted by HFD is clearly shown in Figure 2B (see open bar 1 versus bar 3 in panels a, b, and c). It is important to note that treatment of LFD-*Thrb^PV/PV^Pten*^+/−^mice with metformin reverted tumor progression from vascular invasion (Figure 2A, panel b) to hyperplasia (panel d). More impressively, metformin markedly reverted the tumor phenotypes from vascular invasion (Figure 2A, panel e) and anaplasia (panel f) to hyperplasia (panels g and h) in HFD-*Thrb^PV/PV^Pten*^+/−^mice. The beneficial effects of metformin on the pathological changes are clearly evident in Figure 2B in that no occurrences of vascular invasion and anaplasia were detected in HFD-*Thrb^PV/PV^Pten*^+/−^mice treated with metformin (compare bar 4 with bar 3 in panels b and c). These results indicate that metformin treatment was effective in blocking tumor progression.

### Metformin inhibits the STAT3 signaling pathway in HFD-Thrb^PV/PV^Pten^+/−^mice

**Figure 3:**
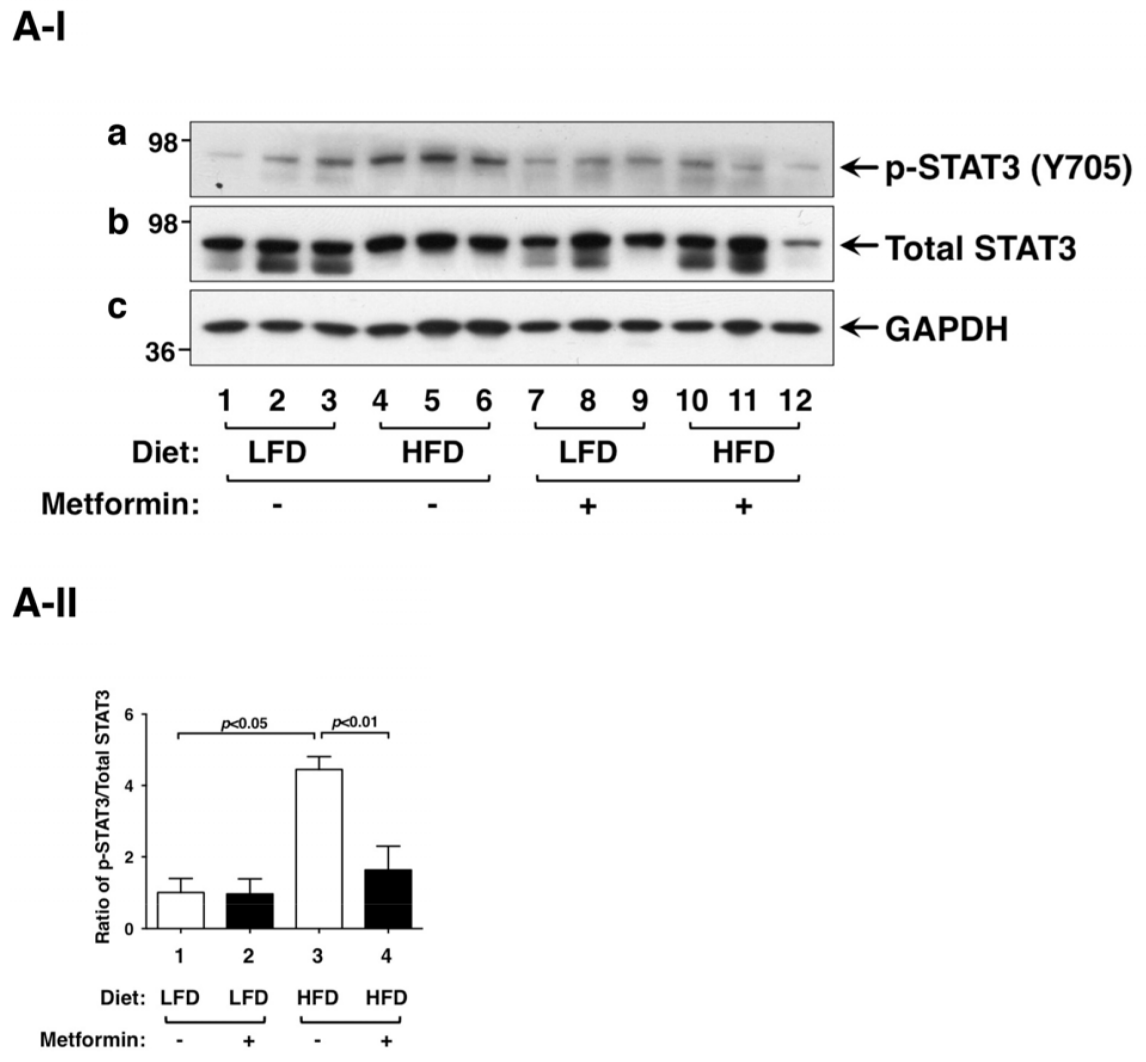
Effect of metformin on STAT3 and p-STAT3 protein abundance in the thyroids of LFD- or HFD- *Thrb^PV/PV^ Pten*^+/−^mice **A-I.** Western blot analysis of protein abundance of phosphorylated-STAT3 (Y705), total-STAT3, and GAPDH as a loading control of thyroid tumors from LFD- or HFD-*Thrb^PV/PV^Pten*^+/−^mice treated with vehicle or metformin as indicated. **A-II.** The band intensities of the protein detected in (A-I) were quantified and compared. The data, shown as mean ± SE, were analyzed by Student's t test. **B-I.** Immunohistochemical analysis (IHC) of p-STAT3 in the thyroids of LFD- or HFD-*Thrb^PV/PV^Pten* ^+/−^mice. IHC was analyzed according to Methods and Materials. Thyroid sections from the vehicle-treated LFD (panel b), metformin-treated LFD (panel d), vehicle-treated HFD (panel f), and metformin-treated HFD (panel h) of *Thrb^PV/PV^Pten* ^+/−^mice were analyzed. The negative controls with no primary antibodies are shown in the corresponding panels (LFD: a and c; HFD: e and g). **B-II.** The p-STAT3-positively stained cells were counted and the data are expressed as percentage of p-STAT3-positive cells versus total cells. The data are expressed as mean±SE (n= 3 slides). The p values are shown.

Previously, we elucidated that the activation of leptin-JAK2-STAT3 signaling accounts for the HFD-induced promotion of thyroid tumor progression in *Thrb^PV/PV^Pten*^+/−^mice [9]. To understand how metformin blocked HFD-induced tumor progression, we first evaluated the changes brought about by metformin in the key regulators of the Leptin-JAK2-STAT3 signaling. As shown in Figure 3A-I, metform in lowered p-STAT3 (Y705) protein levels in thyroid tumors of HFD-*Thrb^PV/PV^Pten*^+/−^mice (compare lanes 10-12 to lanes 4-6) without changing the total STAT3 protein levels (panel b). The quantitative analysis of the ratios of p-STAT3 (Y705) versus total STAT3 indicated that the HFD-induced activation of STAT3 signaling (Figure 3A-II, bar 3) was attenuated by metformin (Figure 3A-II, bar 4). We further carried out immunohistochemical analysis to determine the protein abundance of p-STAT3 (Y705) in thyroid tumor cells in *Thrb^PV/PV^Pten*^+/−^mice. Consistent with the results from western blot analysis, metformin didnot affect the protein levels of p-STAT3 in the tumor cells of LFD-*Thrb^PV/PV^Pten*^+/−^mice (Figure 3B-I, panel d versus panel b; also see the quantitative data: Figure 3B-II, bar 2 versus bar 1). In contrast, p-STAT3 signals clearly had higher intensity in vehicle-treated HFD-*Thrb^PV/PV^Pten*^+/−^mice than in LFD-*Thrb^PV/PV^Pten*^+/−^mice (Figure 3B-I, compare panel f with panel b; Figure 3B-II, bar 3 versus bar 1). Metformin treatment reduced the p-STAT3 signals in thyroid tumor cells of HFD-*Thrb^PV/PV^Pten*^+/−^mice (Figure 3B-I, compare panel h with panel f; also see the quantitative data: Figure 3B-II, bar 2 versus bar 1). Taken together, these data indicate that metformin acted to inhibit the activation of STAT3.

**Figure 4:**
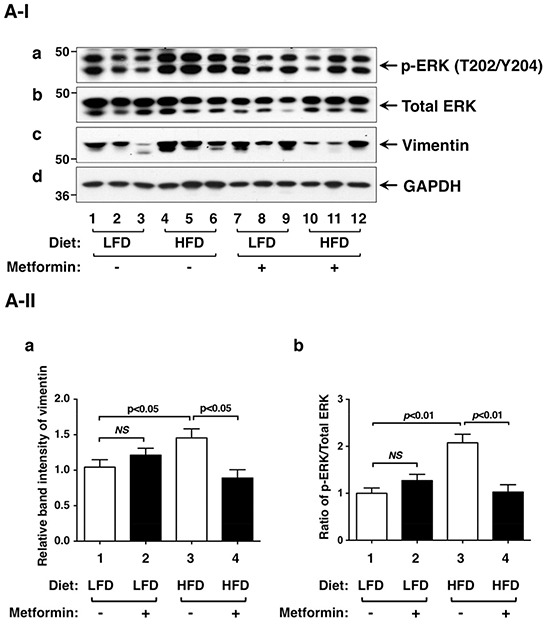
Effects of metformin on protein levels of ERK, vimentin and key regulators of integrin signaling pathway in the thyroids of LFD- or HFD- *Thrb^PV/PV^ Pten*^+/−^mice **A-I.** Western blot analyses of protein abundance of phosphorylated-ERK (T202/Y204), total-ERK, vimentin, and GAPDH as a loading control from LFD- or HFD-*Thrb^PV/PV^Pten*^+/−^mice treated with vehicle or metformin were carried out as described in Materials and Methods. **A-II.** The band intensities of the protein detected in (A-I) were quantified and compared. The data, shown as mean ± SE, were analyzed by Student's t test. **B-I.** Western blot analysis of protein abundance of fibronectin, integrin α6, integrin β1, integrin β3, and GAPDH as a loading control. **B-II.** The band intensities of the protein detected in (B-I) were quantified and compared. The data, shown as mean ± SE, were analyzed by Student's t test.

Leptin mediates its effects not only via STAT3, but also via extracellular signal-regulated kinase (ERK) [28]. We therefore evaluated the activity of ERK by examining p-ERK protein levels. HFD elevated p-ERK (T202/204) without significant changes in total ERK protein levels (Figure 4A-I-a and 4A-I-b, lanes 4-6 versus lanes 1-3; also see the quantitative data: Figure 4A-II-a, bar 3 versus bar 1). Metformin treatment reduced p-ERK protein levels (Figure 4A-I, lanes 10-12 versus lanes 4-6; also see the quantitative data: Figure 4A-II-a, bar 4 versus bar 3). Therefore, leptin-mediated activation of STAT3 and ERK pathways was attenuated by metformin.

Since metformin blocked thyroid tumor progression of HFD-*Thrb^PV/PV^Pten*^+/−^mice, we next focused on the analysis of regulators affecting cytoskeletal structure, cell motility, and migration. We first examined whether vimentin protein levels were affected by metformin. Vimentin is a type III intermediate filament protein and is a major cytoskeletal component in mesenchymal cells. Vimentin is often used as a marker for cells undergoing epithelial-mesenchymal-transition (EMT) during metastatic progression. Vimentin is positively regulated by STAT3 [29, 30]. Moreover, biochemical analyses demonstrated direct interaction of vimentin with ERK, which promoted ERK activation and enhanced vimentin transcription [31, 32]. In line with these findings, we found that elevated vimentin in thyroid tumors of HFD-*Thrb^PV/PV^Pten*^+/−^ mice was inhibited by metformin, thereby decreasing the extent of EMT (Figure 4A-I, panel c, lanes 10-12 versus lanes 4-6; also quantitative data: Figure 4A-II-b, bar 4 versus bar 3) to suppress cell invasion.

We next evaluated whether extracellular matrix components were affected by metformin in thyroid tumor cells of HFD-*Thrb^PV/PV^Pten*^+/−^mice. Fibronectin (FN) is a high molecular weight protein of the extracellular matrix that binds to membrane-spanning receptor proteins, known as integrins. FN plays a major role in cell adhesion, migration, and metastasis [33, 34]. Recent studies suggest that metformin treatment could reduce tumor cell invasion in pancreatic cancer [35]. Metformin treatment of diabetetic patients is associated with low recurrence of cervical lymph node metastasis of differentiated thyroid cancer [36]. Indeed, we found that FN protein abundance was higher in thyroid tumors of HFD-*Thrb^PV/PV^Pten*^+/−^mice than in LFD-*Thrb^PV/PV^Pten*^+/−^mice (Figure 4B-I, panel a, lanes 4-6 versus lanes 1-3; quantitative data: Figure 4B-II-a, bar 3 versus bar 1). Metformin treatment lowered FN protein abundance in thyroid tumor cells of HFD-*Thrb^PV/PV^Pten*^+/−^mice (Figure 4B-I, panel a, lanes 10-12 versus lanes 4-6; quantitative data: Figure 4B-II-a, bar 4 versus 2).

Increased integrin expression levels contribute to earlier metastatic potential of thyroid cancer [37]. For example, integrin α6 has been reported to mediate progression of papillary thyroid cancer [38]. Therefore, we further evaluated the protein levels of FN receptors such as integrins α6, β1, and β3 in thyroid tumors of LFD- or HFD-*Thrb^PV/PV^Pten*^+/−^mice. We found that integrin α6 (Figure 4B-I, panel b), integrin β1 (panel c), and integrin β3 (panel d) were similarly elevated as FN in the thyroid tumors of HFD-*Thrb^PV/PV^Pten*^+/−^mice (Figure 4B-I, lanes 4-6 versus lanes 1-3; quantitative data: Figure 4B-II-b, 4B-II-c, and 4B-II-d, bar 3 versus bar 1). Metformin similarly suppressed the protein levels of integrin α6 (panel b), integrin β1 (panel c), and integrin β3 (panel d) (Figure 4B-I, lanes 10-12 versus lanes 4-6; quantitative data: Figure 4B-II-b, 4B-II-c, and 4B-II-d, bar 3 versus bar 1). Taken together, these results indicate that metformin treatment was effective in attenuating HFD-activated EMT and the FN-integrin signaling pathway to suppress invasion and anaplasia of tumor cells in HFD-*Thrb^PV/PV^Pten*^+/−^mice.

## DISCUSSION

In the present studies, we used the preclinical model of *Thrb^PV/PV^Pten*^+/−^mice fed with HFD to test the effect of metformin on obesity-activated thyroid cancer progression. Treatment of HFD-*Thrb^PV/PV^Pten*^+/−^mice with metformin for 20 weeks had no effect on the survival or the thyroid tumor growth of these mice. Remarkably, however, the treatment reduced the occurrence of capsular invasion and abrogated the development of vascular invasion and anaplasia. Since the endpoint of metformin treatment was 20 weeks, which did not allow sufficient time for metastasis to occur, we were unable to evaluate the effect of metformin on the development of metastasis. Using molecular and biochemical analyses, we found that metformin attenuated the activity of STAT3-ERK-vimentin and FN-integrin signaling to reduce the occurrence of capsular invasion and blocked vascular invasion and anaplasia. However, the altered FN-integrin signaling may not be the only pathway that led to the reduced tumor cell invasion by metformin. We found that metformin also suppressed the occurrence of thyroid tumor cell invasion in LFD-treated *Thrb^PV/PV^Pten*^+/−^mice, but no changes in the FN-integrin signaling. These observations suggested that there were additional pathways affected by metformin that could reduce the tumor cells invasion. The identification of such pathways would await future studies.

The findings that metformin blocked vascular invasion of thyroid tumors of *Thrb^PV/PV^Pten*^+/−^mice are in line with the reports in which the anti-angiogenic effects of metformin were described in colon cancer [39], HER2+ tumor cells [40] and breast tumors [41, 42]. In colon cancer, metformin was shown to down regulate tumor angiogenesis and augment the antitumor effect oxaliplatin [39]. In HER2+ tumor cells, metformin treatment decreased microvessel-induced inhibition of tumor angiogenesis [40]. In breast tumors, metformin reduced tumor microvessel density and attenuated tumor angiogenesis [41, 42]. While metformin was shown to have anti-neoplastic effects in diabetic patients with differentiated thyroid cancer [25], whether metformin has anti-angiogenic effects in thyroid cancer progression remains to be elucidated.

That metformin could block cancer cell invasion and anaplasia, but not tumor growth, suggested that the molecular pathways of anti-diabetic and anticancer actions of metformin could differ *in vivo.* Although the detailed molecular basis underlying the metabolic effects of metformin are not completely understood, the primary molecular mechanism mediating this effect appears to be the activation of AMP-activated protein kinase (AMPK) and the subsequent inhibition of mammalian targets of rapamycin (mTOR) [43–45]. Intriguingly, no significant changes in the AMPK-mTOR-p70^S6K^ and AMPK-mTOR-4EBP1 signaling pathways were detected in thyroid tumors of HFD-*Thrb^PV/PV^Pten*^+/−^mice (data not shown). These results suggest that metformin treatment had not led to the changes of protein synthesis and lipid metabolism during thyroid carcinogenesis of HFD-*Thrb^PV/PV^Pten*^+/−^mice as expected for its anti-diabetic effects. This notion is consistent with our findings that metformin treatment did not affect tumor growth (Figure 1B-b). We have shown previously that in the thyroid of *Thrb^PV/PV^Pten*^+/−^mice, PI3K-AKT is highly activated to drive aggressive tumor growth [8]. Knowing that AKT is also a downstream target of activated insulin signaling [46], we speculate that highly activated AKT signaling could interfere with the AMPK signaling via cross talk such that the metformin-mediated inhibition of AMPK could be blunted in the thyroid of HFD-*Thrb^PV/PV^Pten*^+/−^mice. This conjecture raises the possibility that the effectiveness of metformin as an antiproliferative drug would depend on the genetic abnormalities and altered signaling pathways of thyroid cancer. That AMPK sensitivity to metformin inhibition could be modulated by cellular context is not without precedent. It has been shown that p53 is involved in mediating the energy-conserving response to AMPK activation and that loss of p53 heightens the metformin-induced energy stress on cancer cells, implying that metformin may have increased efficacy in p53-deficient tumors like ovarian cancer [47]. In addition, recent studies showed that cancer cell lines with mutations in mitochondrial DNA genes have an increased response to metformin [48]. In line with these observations, an ongoing clinical trial is currently evaluating the effect of metformin on dosing of levothyroxine (T4) required for TSH suppression in patients with differentiated thyroid cancer (ClinicalTrials.gov; Identifier: NCT01341886). The outcome of this trial should uncover how the sensitivity of TSH response to T4 is modulated by metformin and could further support the notion that the effectiveness of metformin is subject to modulation by cellular regulators.

The present studies showed that metformin failed to exert antiproliferative effects to inhibit HFD-induced tumor growth in *Thrb^PV/PV^Pten*^+/−^mice. Still, metformin was effective in delaying thyroid cancer progression by reducing the frequency of capsular invasion and abrogating the development of vascular invasion and anaplasia. These findings suggest that metformin would be useful in preventing the metastatic spread of thyroid cancer. These findings also suggest that metformin would be useful as an adjuvant in combination treatment with other antiproliferative drugs. In view of metformin's safety and affordability, we would expect that clinical trials could soon be extended to test the effectiveness of metformin to treat differentiated and de-differentiated thyroid cancer. In addition to *Thrb^PV/PV^Pten*^+/−^mice, other mouse models have recently been developed to study thyroid cancer [49–54], and it would be possible to use different mouse models mimicking different types of thyroid cancer to assess the effectiveness of metformin in those models. Furthermore, these preclinical studies might also lead to the identification of genes that could potentially enhance metformin's effects, as well as genes that could render metformin insensitive. The findings of these proposed preclinical studies would be very helpful in the consideration of using metformin to improve the outcome of patients with thyroid cancer.

## MATERIALS AND METHODS

### Mice and treatment

The National Cancer Institute Animal Care and Use Committee approved the protocols for animal care and handling in the present study. Mice harboring the *ThrbPV* gene (*Thrb^PV/PV^*mice) were prepared via homologous recombination, and genotyping was carried out using the PCR method, as previously described [8]. *Pten*^+/−^mice were kindly provided by Dr. Ramon Parsons (Columbia University, NY, USA). *Thrb^PV/PV^Pten*^+/−^mice were obtained by first crossing *Pten*^+/−^mice with *Thrb^PV/PV^*mice followed by further crossing the heterozygous offspring *Thrb^PV/+^Pten*^+/−^ with *Thrb^PV/+^Pten*^+/−^mice. The high fat diet (HFD) (60 % Kcal from fat) was purchased from Research Diets (New Brunswick, NJ, USA). The mice, housed in SPF (specific pathogens free) animal facility, were administrated HFD diet from the age of 6 weeks until the end of the study. Metformin (cat#M1566, Spectrum, Gardena, CA, USA) was diluted in drinking water (0.5 mg/ml) [55], and the solution was changed weekly. They were monitored until they reached the age of 21 weeks, or they became moribund with rapid weight loss, hunched posture, and labored breathing. After the mice were euthanized, the thyroids were dissected for weighing, histologic analysis, and biochemical studies.

### Histopathologic analysis

Thyroid glands, lungs and inguinal fat were dissected and fixed in 10% neutral-buffered formalin (Sigma-Aldrich, St. Louis, MO, USA) and subsequently embedded in paraffin. Five-micrometer-thick sections were prepared and stained with hematoxylin and eosin. For each animal, single random sections of thyroid were examined. For thyroids, morphologic evidence of hyperplasia, capsular invasion, and vascular invasion was routinely examined in that single section.

Immunohistochemistry (IHC) was conducted as previously described with some modifications [50]. For the antigen retrieval step, slides were heated in 0.05% citraconic anhydride solution (pH 7.4; Sigma-Aldrich, St. Louis, MO, USA) at 98°C for 60 minutes followed by treatment with rabbit anti-p-STAT3 antibody (1:100 dilution, Cell Signaling, Denver, MA, USA) and anti-cleaved caspase 3 antibody (dilution 1:300, cat. 9661, Cell Signaling) at 4°C overnight. The antigen signals were detected by treatment with the peroxidase substrate diaminobenzidine, followed by counterstaining with Gill's hematoxylin (Electron Microscopy Sciences, Hatfield, PA, USA). Relative positive cell ratio was quantified by using NIH IMAGE software (Image J 1.47).

### Western blot analysis

Preparation of whole-cell lysates from thyroid glands has been described previously [50]. The protein sample (30 μg) was loaded and separated by SDS-PAGE. After electrophoresis, the protein was electrotransferred to a poly vinylidenedifluoride membrane (Immobilon-P; Millipore Corp., Billeria, MA, USA). The antibodies phosphorylated STAT3 (1:500 dilution), total-STAT3 (1:1,000 dilution), p-ERK (1:500 dilution), total-ERK (1:1,000 dilution), vimentin (1:1,000 dilution), and GAPDH (1:1,000 dilution) were purchased from Cell Signaling Technology (Danvers, MA, USA). Antibodies for fibronectin (1:200 dilution), integrin α6 (1:200 dilution), integrin β1 (1:200 dilution), integrin and β3 (1:200 dilution) were purchased from Santa Cruz Biotechnology (Dallas, TX, USA). The blots were stripped with Re-Blot Plus (Millipore, Billeria, MA, USA) and reprobed with rabbit polyclonal antibodies to GAPDH. Band intensities were quantified by using NIH IMAGE software (Image J 1.47).

### Statistical analysis

All data are expressed as mean ± standard errors, and Student's t test was used to compare continuous variables accordingly. The Kaplan–Meier method with log-rank test was used to compare survival in each treatment group. Statistical significance was set at p<0.05. GraphPad Prism 6.0 (GraphPad Software, La Jolla, CA, USA) was used to draw graphs.
